# Triple Tooth in Primary Dentition: A Rare Case Report

**DOI:** 10.3390/children12040395

**Published:** 2025-03-21

**Authors:** Maria Teresa Xavier, Sara Rosa, Ana Daniela Soares, Inês Nunes, Bárbara Cunha, Ana Luísa Costa

**Affiliations:** 1 Institute of Paediatric and Preventive Dentistry, Faculty of Medicine, University of Coimbra, 3000-075 Coimbra, Portugal; saramfrosa@sapo.pt (S.R.); ana.dani.soares@gmail.com (A.D.S.); inesafnunes@gmail.com (I.N.); barbaracunha01@gmail.com (B.C.); aluisacosta@sapo.pt (A.L.C.); 2 Center for Innovation and Research in Oral Sciences (CIROS), Faculty of Medicine, University of Coimbra, 3000-075 Coimbra, Portugal

**Keywords:** children, fusion, gemination, primary teeth, tooth abnormalities, triple teeth, triplication, supernumerary tooth

## Abstract

The occurrence of triplication in the deciduous teeth is rare. However, it can cause several problems in primary dentition, alteration of development, and eruption of permanent successors. **Case Presentation**: A three-year-old boy presented with an exuberant acute periapical abscess in the left front teeth region of the upper jaw. Examination revealed a presence of a triple tooth involving the central and lateral left primary incisors and a supernumerary tooth. Radiographs showed that the fused teeth had separate roots, pulp chambers and root canals. The implemented treatment was extraction under local anesthesia. After 2 years of observation, surgical exposure of the crowns of the permanent maxillary central incisor was performed. After seven years, the permanent dentition was completed without any sequelae. **Discussion**: Triple tooth, as observed in this case report, results from the union of three tooth germs, potentially influenced by physical, hereditary, or environmental factors, leading to esthetic and functional issues and increased susceptibility to caries. Treatment is challenging, requiring preventive care, complex endodontic procedures, and, in some cases, extraction with space maintenance to avoid future orthodontic complications. **Conclusion:** Early diagnosis, an adequate treatment plan and clinical monitoring should be performed, aiming at preventing the possible disturbances.

## 1. Introduction

Dental anomalies such as fusion, gemination, supernumerary teeth, and concrescence are not rare [1,2]. Although “double teeth” are relatively common, with a reported prevalence of 1% to 1.55%, the fusion of three teeth, known as a “triple tooth”, is extremely rare. The prevalence of triplication in the deciduous dentition has been reported to be 0.02% [1,3]. This condition occurs in the maxillary arch three times more often than in the mandibular arch, with a documented case ratio of 22:7. Additionally, it is more commonly observed in males [2,3].

Fused teeth result from the union of two or three normal tooth germs, a condition known as synodontia, or from the union of one or two normal tooth germs with a supernumerary tooth. The fusion can be complete or incomplete, depending on the timing during tooth development. In complete fusion, which occurs early, the pulp chamber and root canal may be joined, whereas in incomplete fusion, which occurs later, these structures may remain separate. When fusion takes place after the crowns are fully formed, the teeth are joined only by the cementum, a condition called concrescence [4,5].

Gemination occurs when an attempted tooth-germ cleavage fails, leading to the incomplete tooth formation of two teeth that typically involve one pulp chamber, a single root, and a common pulp canal. Twinning, on the other hand, refers to the complete formation of two nearly identical teeth—one normal and one supernumerary—that remain fused, usually with a single root and pulp canal. In cases involving three tooth entities, the terms “triple tooth”, “triple teeth”, or “triplication” are used, covering all types of unions. The clinical presentation of fusion and gemination, whether involving normal or supernumerary teeth, appears similar [4,5].

The first description of ‘three fused teeth’ was by Grossman in 1980, whereas the term ‘triple tooth’ was first used by Knapp and McMahon in 1983 [6]. Fusion of three teeth, a rare dental anomaly with the prevalence of 0.02%, is defined as triplication [7,8]. Triplication can result from fusion, gemination, concrescence, or a combination of gemination and fusion, typically involving the union of two primary teeth and a supernumerary tooth [3,9]. It is classified into two categories: type I and II. Type I involves fusion with three pulp chambers and three root canals and is further divided into subtypes: type IA, which is the fusion of two normal teeth and a supernumerary tooth, and type IB, which is the fusion of three normal teeth. Type II involves fusion with two pulp chambers and two root canals and also has two subtypes: type IIA, referring to the fusion of one geminated tooth and a supernumerary tooth, and type IIB, which is the fusion of a geminated tooth and a normal tooth [3]. The clinical and radiographic appearance varies depending on the stage of tooth development at the time of fusion [10]. Nowadays, the preliminary diagnosis can be confirmed under CBCT analysis, and the separate pulp chambers may be observed and the pulp chamber transforming into one unified in a single chamber is identified [11].

This paper aimed to report a rare case of a young patient presenting large triple teeth in the left maxillary incisor region and further discuss the importance of an early diagnosis and comprehensive treatment plan.

## 2. Detailed Case Description

**Figure 1:**
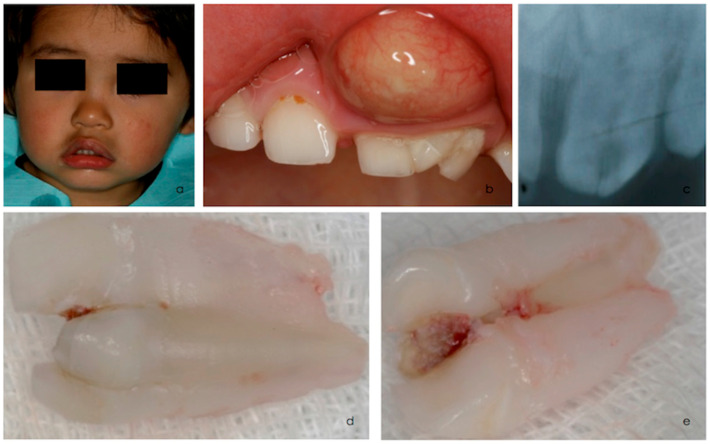
(**a**–**c**). Extraoral and intraoral exam. Intraoral periapical radiograph shows fusion of two primary incisors with supernumerary tooth, with separate pulp chamber and root canals. Based on the clinical and radiographic findings, the diagnosis of triple teeth was confirmed. (**d**,**e**) The treatment plan was aimed to extract the offending tooth with local anesthesia, under antibiotic prescription.

A 3-year-old boy was referred to the University Dental Clinic due to an acute periapical abscess in the left front teeth region of the upper jaw, face edema and pain (Figure 1a).

Extraoral and intraoral examinations revealed an abscess associated with a deep proximal carious lesion affecting a fused maxillary left central and lateral incisor with a supernumerary tooth (Figure 1a,b). Clinical examination confirmed triplication involving the left primary central and lateral incisors and a supernumerary tooth (Figure 1b). The triple tooth was larger in the mesiodistal dimension, had an irregular morphology, and presented a deep caries lesion on the palatine surface associated with necrosis. These teeth exhibited neither mobility nor fractures. A periapical radiograph of the maxillary anterior region suggested the fusion of three separate teeth (Figure 1c). The presence of the central and lateral permanent incisors was confirmed (Figure 1c).

After abscess drainage and antibiotic therapy, due to the difficulty in performing root canal treatment on such teeth and the young patient’s limited cooperation, extraction under periapical infiltrative anesthesia with lidocaine 2% with epinephrine 1:80,000 of the triple teeth was planned and subsequently performed (Figure 1d,e). Hemostasis was successfully achieved, and post-extraction instructions were provided to the parents. A 5% sodium fluoride varnish (Duraphat, Colgate-Palmolive, New York, NY, USA) was applied every 6 months. Oral hygiene instructions were given to both the patient and the parents.

To prevent functional, esthetic and phonetic issues, the missing teeth should have been replaced with a transitional partial denture or another type of space maintainer. However, due to the child’s limited cooperation, this was not possible at that stage.

**Figure 2:**
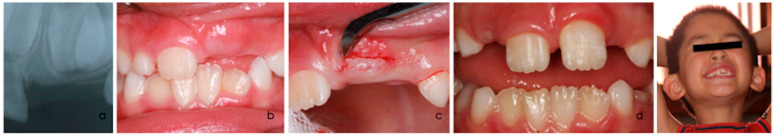
(**a**–**c**) Clinical and radiographic exam from the two-year follow-up and a delayed eruption of the permanent central was observed. (**d**,**e**) An ulectomy was performed and the upper central incisor erupted 5 months later; extraoral and intraoral exam.

**Figure 3:**
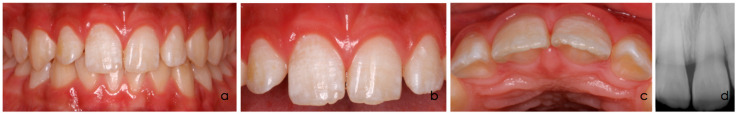
(**a**–**d**) Clinical appearance and radiographic exam of the permanent dentition at the seven-year recall.

Annual follow-ups were conducted to monitor the clinical and radiographic eruption of the permanent successors. A delayed eruption of the permanent central incisor was observed. At the two-year follow-up, an ulectomy was performed to facilitate the eruption of the left central permanent incisor (Figure 2a–c) and, five months later, the tooth had fully erupted (Figure 2d,e). After seven years follow-up, the permanent teeth had erupted without complications (Figure 3a–d).

The author(s) have obtained written informed consent from the patient for the publication of the case report details and associated images.

## 3. Discussion

In this case report, the triple tooth resulted from fusion, which is defined as the embryological union of two or more separate buds with confluence of dentin [2,9]. While the exact cause of fusion is still unclear, it is believed that physical forces or pressure on developing tooth germs may cause to the union of the enamel organ and the dental papilla, resulting in the fusion of teeth. Some authors propose that hereditary factors, excessive vitamin A intake, viral infection, or thalidomide use during pregnancy could be potential contributing factors [12,13,14].

Fusion may also be linked to reduced available space caused by the presence of a supernumerary tooth and the proximity of tooth germs. This indicates a potential common underlying factor associated with dental lamina hyperactivity. Recent studies have shown that Notch signaling, mediated through the Jagged2 gene, plays a crucial role in tooth development and fusion [15,16]. Clinically, the appearance of gemination and fusion involving normal or supernumerary teeth is identical. However, they can be differentiated by dental formula: there is generally a difference in the dental formula, as fusion typically results in a reduced tooth count, whereas gemination maintains a normal count when the gemination tooth is considered as one [9]. Radiographically, geminated teeth generally have a single root with one root canal [9], whereas fused teeth often have separate pulp chambers and root canals [3].

The triple tooth exhibits a distinct clinical presentation, with a labial supernumerary tooth fused to two normal teeth—the central incisor on the mesial side and the lateral incisor on the distal side. Notably, only the central and lateral incisors are affected, which differs from the diverse presentations observed in cases of double teeth [17].

The precise diagnosis of a triple tooth using CBCT improves treatment planning, assisting in the decision between extraction and preservation, particularly when endodontic treatment is required. In the present case, since extraction was the chosen treatment option and in adherence to the ALARA principle, CBCT was not performed. However, this imaging modality could have provided valuable information regarding pulp anatomy, root complex vertical grooves, and the site of pulp fusion and could be considered a limitation of this case report.

In this case, no other dental anomalies were detected in the primary or permanent dentition, and no other anomalies were observed, except a mild fluorosis. These factors, along with the consistent incisor location of triple teeth, suggest that the abnormality is singular and confined to the anterior segment of the dental lamina [18].

Triple teeth cause esthetic, functional and clinical issues in primary dentition. They are more susceptible to dental caries due to deep grooves at the fusion site of union, which are difficult to clean. Additional complications include fractures, periodontal disease (if a fissure on the buccolingual side is prolonged to the root surface), abscesses, fistulas, arch asymmetry, and occlusal disturbances [2]. Delayed exfoliation and permanent dentition anomalies—such as impaction, delayed or ectopic eruption, supernumerary teeth or missing teeth, root dilacerations and root resorption—have also been described [1,2,9]. Therefore, a close follow-up is of major importance until the permanent teeth fully erupt. To our knowledge, this article presents the longest follow-up of 7 years.

Treatment is always challenging due to factors such as tooth size, pulp size, and surface proximity caused by deep grooves, along with the atypical crown morphology. These complexities make endodontic, surgical, and restorative procedures more difficult, requiring an approach tailored to the clinical situation. The child’s esthetics often play a key role in the decision to retain or extract the affected teeth, depending on the dentition stage (deciduous, mixed, or permanent), the presence of small or deep carious lesions, the restorability of the teeth, and the pulp status [9]. Unfortunately, a conservative approach was not feasible in this case, as the triple tooth was necrotic, and the complexity of the root canal system rendered effective root canal treatment nearly impossible. In case of extraction, a space maintainer or other transitional partial denture maybe an option to improve esthetics and phonetics and allow permanent tooth eruption. Monitoring remains the best treatment option, and preventive procedures, such as good oral hygiene, fluoride application and sealants, should be placed in the grooves of the buccal and lingual surfaces to prevent dental caries lesions. In cases of delayed exfoliation, extraction may be more recommended to avoid malocclusion [2,3,19].

## 4. Conclusions

In the primary dentition, number anomalies are usually underestimated, especially when they are asymptomatic. However, their presence may have a marked effect on the permanent dentition as they can indicate future dental anomalies, especially hypodontia, and/or implicate the normal development of dentition. Early diagnosis using proper radiographic examination is crucial to implement preventive and restorative treatments, followed by careful monitoring until exfoliation. Therefore, the treatment of fused teeth should consider the phase of the dentition period to prevent adverse effects and improve the child’s dental health.

The treatment protocol for triple teeth generally starts with observation until exfoliation. Depending on the case, it may involve groove sealant/restoration, periodontal and orthodontic interventions, if necessary, endodontic treatment, or extraction if the pulp is affected.

## Data Availability

The original contributions presented in this study are included in the article. Further inquiries can be directed to the corresponding author.
